# Primary Alveolar Rhabdomyosarcoma of the Breast in an Adult: An Extremely Rare Case

**DOI:** 10.1155/2019/6098747

**Published:** 2019-03-28

**Authors:** Helen J. Trihia, Natasa Novkovic, Ioannis Provatas, Anastasios Mavrogiorgis, Evangelos Lianos

**Affiliations:** ^1^Department of Pathology, Metaxas Memorial Cancer Hospital, Piraeus, Greece; ^2^Department of Pathology, Vostanio Hospital, Mytilini, Greece; ^3^Department of Medical Oncology, Metaxas Memorial Cancer Hospital, Piraeus, Greece

## Abstract

Sarcomas of the breast constitute less than 1% of all malignant breast tumors and primary rhabdomyosarcoma (RMS) is a very rare entity with limited case reports in the literature. RMS is common in children and adolescents and rare in adults. Primary RMS arising from the breast is exceedingly rare in adults. We report a case of a primary RMS of the breast in a 60-year-old woman, who presented in an early stage, mimicking invasive ductal carcinoma clinically and is in complete remission after three years of diagnosis and one year of treatment.

## 1. Introduction

Rhabdomyosarcoma (RMS), the most common pediatric soft tissue tumor, rarely occurs in the adult population. It represents less than 3% of all adult primary soft tissue sarcomas. The breast is an exceedingly rare primary site of occurrence and occurs mainly in children. Till 2007, there were only three cases presenting in adults more or equal to 40 years of age [1–3]. Till now there are nine cases of primary rhabdomyosarcoma of the breast presented in international journals. We present here an additional case, which was treated in our Cancer Institute according to the IRS III (Intergroup Rhabdomyosarcoma Study) protocol.

## 2. Case Presentation/Methods

**Figure 1:**
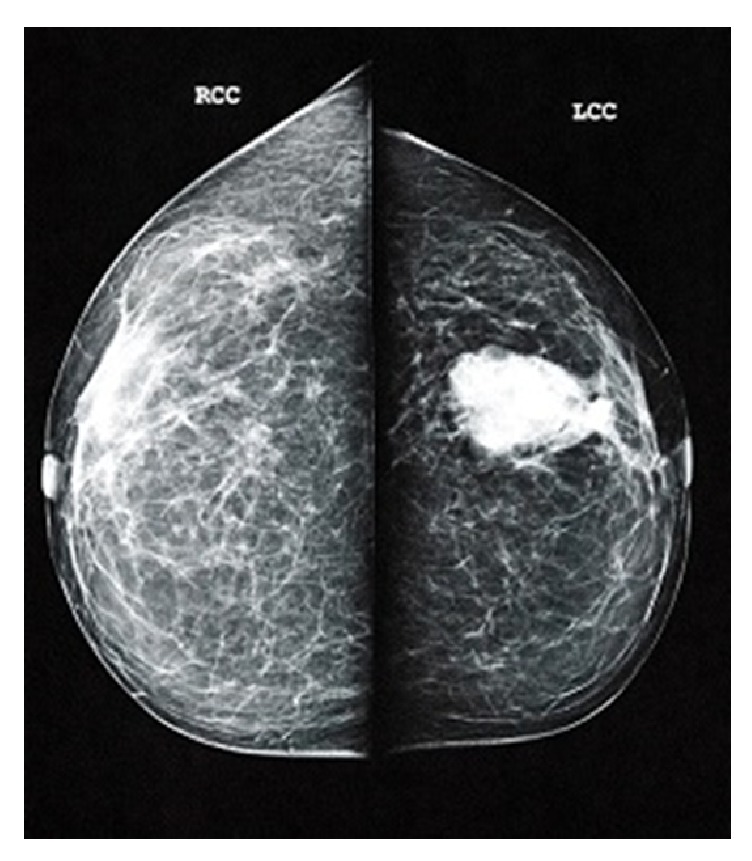
Mammography demonstrates a stable oval, circumscribed mass in the medial part of the left breast.

A 60-year-old woman referred to our medical centre with a 2-month history of an approximately 4 cm lump in the medial part of her left breast. Clinical examination revealed no palpable left axillary lymph nodes. The patient had no other significant medical history. In mammography, a radiologic circumscribed, well-demarcated density, of her medial left breast without lymph node enlargement, was identified (Figure 1). She underwent a fine needle aspiration (FNAC) of the breast lump which suggested a poorly differentiated carcinoma. Metastatic work-up including computed tomography (CT) of the thorax, upper and lower abdomen, revealed no evidence of disease elsewhere. Bone marrow scan was normal. She underwent total mastectomy and sentinel lymph node (SLN) biopsy.

**Figure 2:**
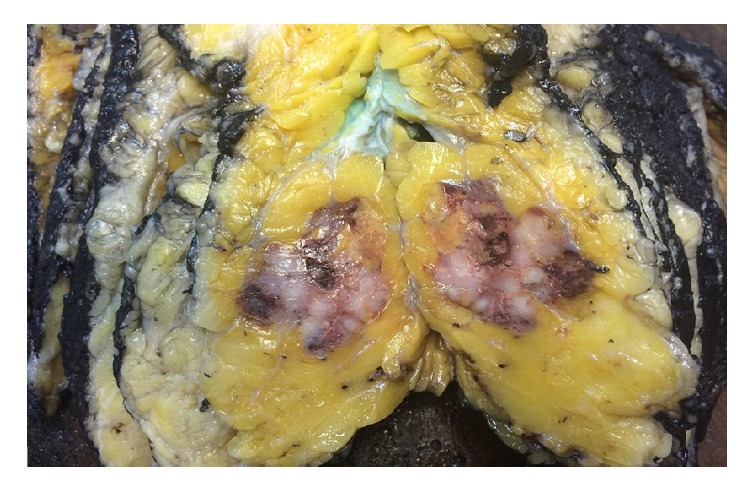
Macroscopic appearance of a well circumscribed, solid with subtle nodularity tumor, with central areas of necrosis.

A 22,5X17X5cm mastectomy specimen and sentinel lymph node biopsy (SLNB) was performed. The SLNB performed during surgery was negative for malignancy. On gross examination, serial sectioning revealed a large solid, well circumscribed, lobulated, with subtle nodularity tumor, measuring 4,8X4X3,8cm, with central necrosis (Figure 2).

**Figure 3:**
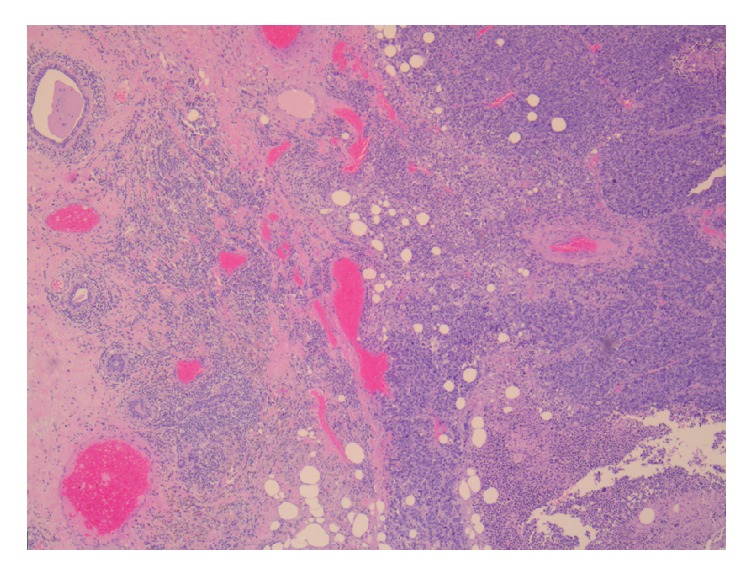
Histological appearance of infiltrative lesion (on the right) of the breast (on the left) composed of sheets of small round blue cells (Hematoxylin & eosin, original magnification X50).

**Figure 4:**
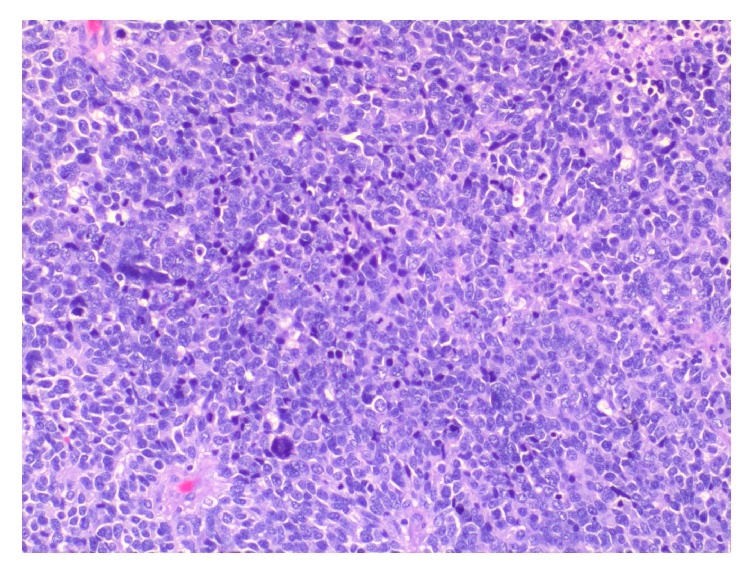
Histological examination shows solid infiltration of small/intermediate neoplastic cells displaying round hyperchromatic pleomorphic nuclei with indistinct cytoplasm and brisk mitotic activity (Hematoxylin & eosin, original magnification X200).

**Figure 5:**
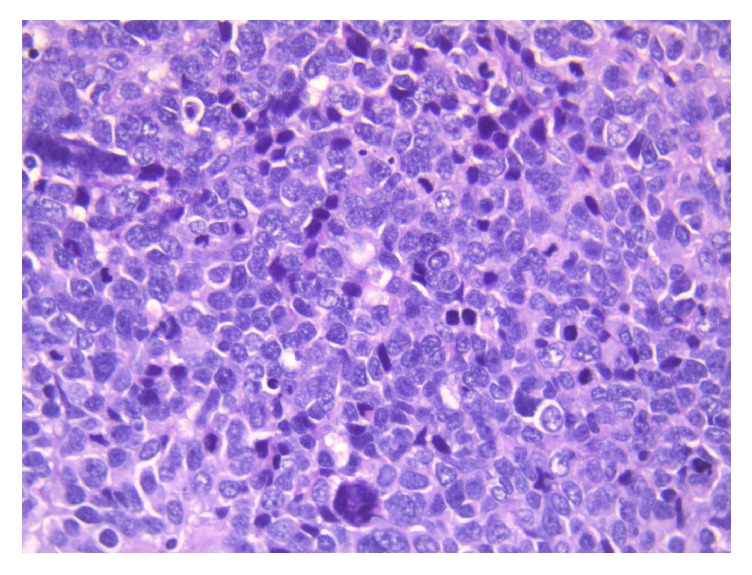
Same as Figure 4, higher magnification.

**Figure 6:**
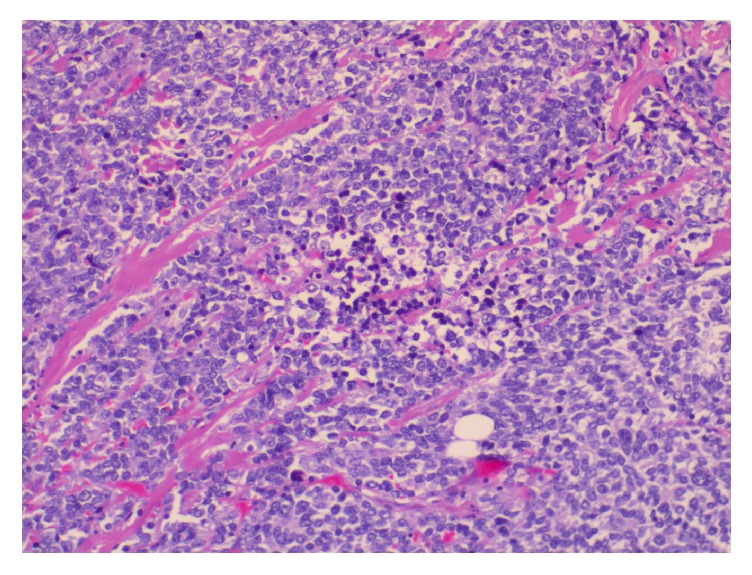
Tumor cells grow in nests separated by hyalinized fibrous septa (X200, H&E stain).

**Figure 7:**
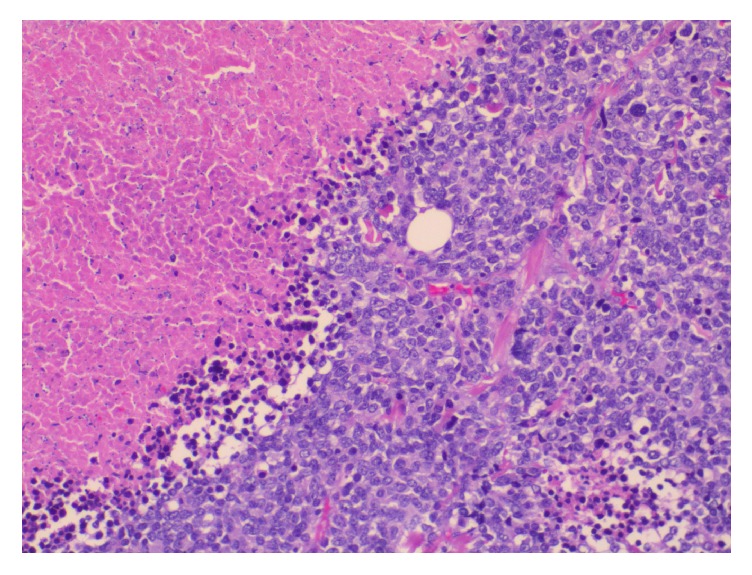
Tumor cells with extensive necrosis (H&E, X200).

**Figure 8:**
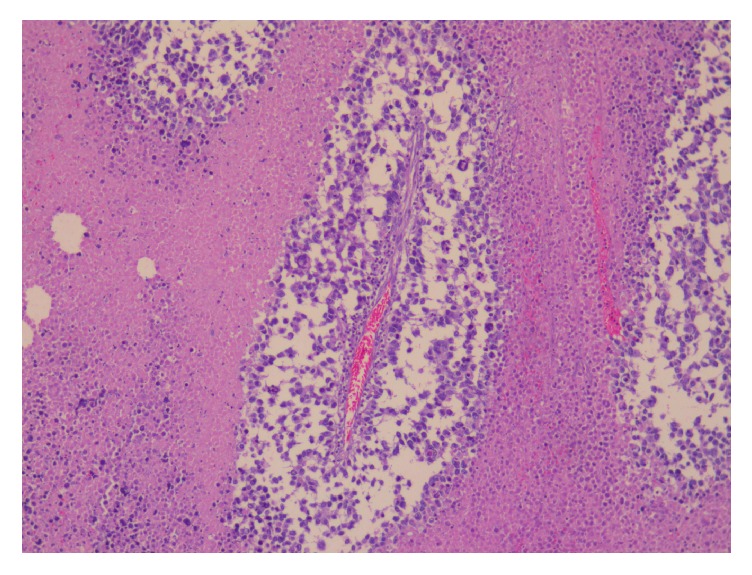
Alveolar pattern with necrosis (H&E, X200).

Surgical specimen was fixed in 10% buffered formalin, routinely processed, and embedded in paraffin. Histological slides of the formalin-fixed tumor tissue (one fragment per centimeter of the tumor was sampled) were deparaffinized and stained with hematoxylin-eosin. The final histopathological report showed an undifferentiated, high grade tumor of the “small round blue cell” morphology, suggestive of rhabdomyosarcoma (Figures 3–8). This was further confirmed by immunohistochemistry (IHC). Immunohistochemistry (IHC) was performed according to manufacturer's protocols and included evaluation of expression of desmin (clone D33; manufacturer DAKO), myogenin (clone F5D; manufacturer DAKO), myo-D1 (clone 58A; manufacturer Immunologic), neurofilament (clone 2F11; manufacturer DAKO) and bcl-2 (clone 124; manufacturer DAKO). Positive and negative controls were used.

**Figure 9:**
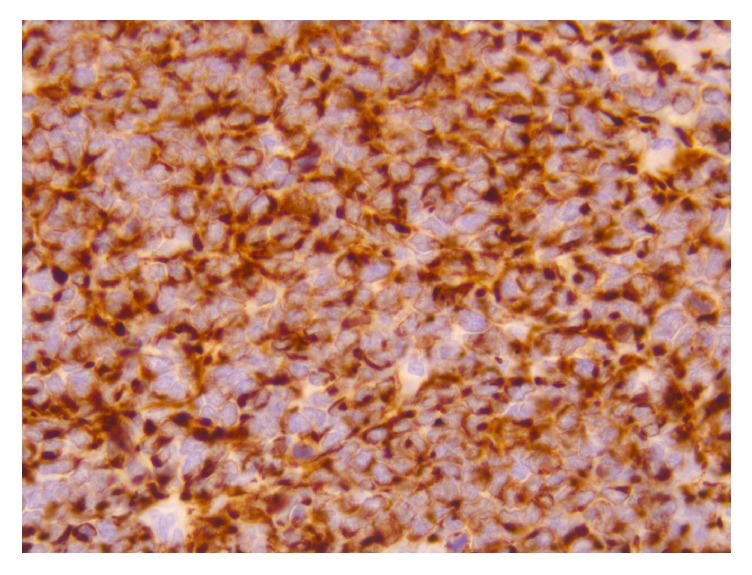
Immunohistochemistry shows positive cytoplasmic (dot-like) staining of the tumor cells for desmin (X200).

**Figure 10:**
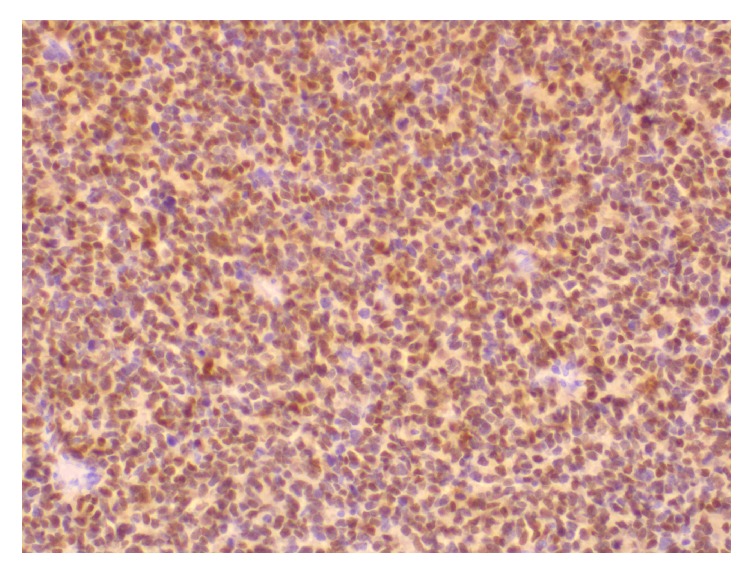
Immunohistochemistry shows positive nuclear staining of the tumor cells for myogenin (X100).

**Figure 11:**
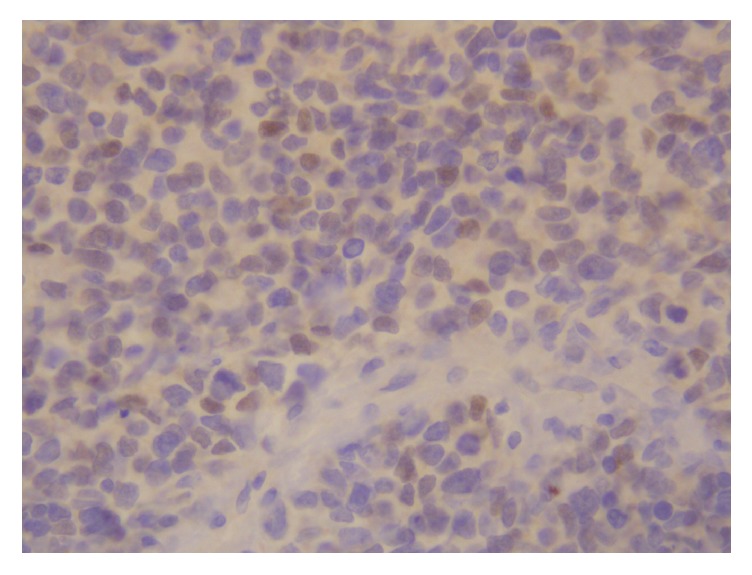
Immunohistochemistry shows positive nuclear staining of the tumor cells for myo-D1 (X200).

**Figure 12:**
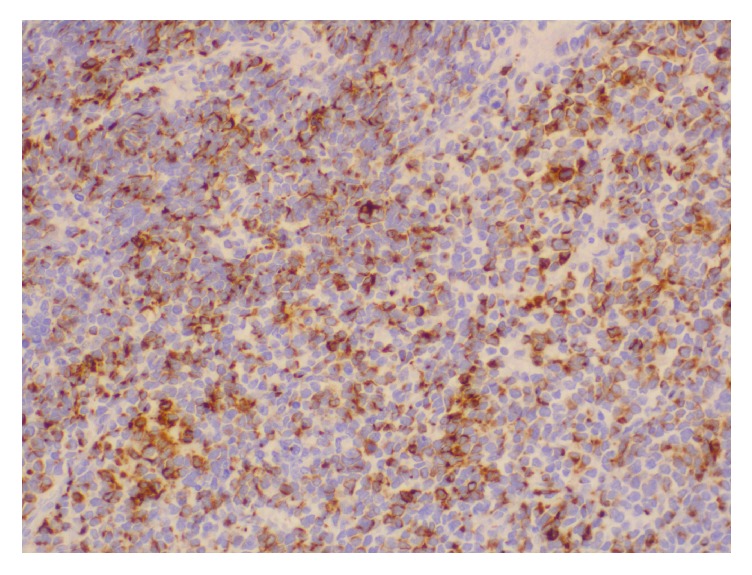
Immunohistochemistry shows positive cytoplasmic (dot-like) staining of the tumor cells for neurofilament (NF) (X100).

**Figure 13:**
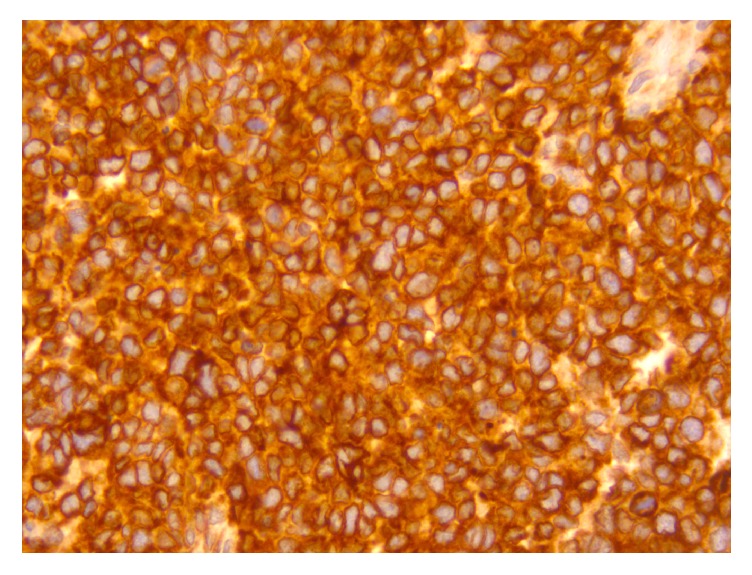
Immunohistochemistry shows positive cytoplasmic staining of the tumor cells for bcl-2 (X400).

Staining for desmin (Figure 9), myogenin (Figure 10), myoD1 (Figure 11), NF (Figure 12), and bcl-2 (Figure 13) confirmed the diagnosis of RMS (solid variant of alveolar type). The tumor was staged as T2 pN0 (sn), according to TNM Classification.

After the exclusion of secondary origin, the woman was treated with chemotherapy based on Intermediate risk-good prognosis Alveolar group I-III. The patient was given 14 cycles of second-line chemotherapy with vincristine, dactinomycin, and endoxan (VAC). The patient is disease free at the follow-up of 24 months from the completion of treatment.

## 3. Discussion

Pure primary rhabdomyosarcoma of the breast is exceedingly rare and occurs mainly in children [4]. It is thought to arise from immature mesenchymal cells that are committed to skeletal muscle lineage, but these tumors can also arise in tissues in which striated muscle is not normally found [5]. RMS comprises the most common soft tissue sarcoma among children and adolescents, related to its linkage with somatic development and these tumors are uncommon in adults [6]. According to previous reports from Intergroup Rhabdomyosarcoma Study Group (IRS) of the United States, only 0.2% of RMS patients diagnosed were of breast origin. On the other hand, when confined to age between 10 and 21 years of age, 1.6% had breast origin RMS [4].

Rhabdomyosarcoma (RMS) may arise from anywhere in the body. Common locations are head and neck, genitourinary trunk and extremities. Common pathologic variants are embryonal and alveolar.

Rhabdomyosarcoma (RMS) may also arise in other sites, such as intrathoracic, perineal-perianal region, biliary tract, liver, brain, trachea, heart, breast, or ovary [5]. Breast involvement is very rare, both as primary or metastatic site [7].

The occurrence of RMS, primary in or metastatic to breast, has been regarded as an uncommon event. Records of 26 patients with diagnoses of breast RMS, primary or secondary, entered in the Intergroup Rhabdomyosarcoma Study (IRS) (1972-1992), were reviewed. Of the 26 IRS cases, the histologic subtype was alveolar in 24, emryonal in 1, and not determined in 1 [4]. All, in young females, with ages ranging from 11.5 to 20.2 years. A review of demographic data and treatment of 24 cases of primary RMs in children and adolescents previously reported in the literature, is reported by Bayramoglu et al. (2018)[8]. There was only one IHC proven case of primary RMS of the breast, presenting after the fourth decade [1]. Rarely, it is reported in adults, with the oldest patient being 60 years old. Evans (1953)[3] reported primary rhabdomyosarcoma of the breast in a 41-year-old woman. Sailer (1937)[9] gave an account of a rhabdomyosarcoma which occurred in the breast of a coloured woman aged 38 years. A case of a 66-year-old woman with two different solid primary cancers (breast and RMS) with synchronous bone marrow metastases has recently been reported in the literature [10]. To the best of our knowledge, there are only nine cases of primary RMS of the breast in adults, reported in international journals [1, 2, 11–16]. A summarizing table of these cases is provided, including ours (Table 1). The women were 30-60 years of age (mean 45). The left breast was involved in five cases, the right breast, in three cases and unknown in two. Three cases were of embryonal type (one of which of spindle cell type), two of alveolar type, two pleomorphic and three unknown.

Cell of origin of RMS is debated. Myogenic RMS may be due to subset of muscle forming cells, called satellite cells. Nonmyogenic RMS may be due to mesenchymal progenitor cells which are committed not only to myogenic lineage but also to produce tissue stromal elements (fat, fibroblasts, connective tissue). It is hypothesized that such cells may circulated in different organs and may give rise to RMS [17].

Heterologous rhabdomyoblastic differentiation in malignant phylloedes tumor or metaplastic carcinoma is more frequent and observed in older women, but is still very uncommon [18, 19].

Among patients with metastatic RMS, synchronous to the breast is seen in 3.7% of cases. The commonest primary sites for metastatic RMS to the breast are extremities and head and neck areas.

Diagnosis of RMS is by detection of cross striations under light or electron microscopy. Staining for actin, desmin, myogenin, and myoD1 confirms the diagnosis. Molecular and genetic markers are also used to differentiate the various subtypes[20].

Fine needle aspiration cytology (FNAC) is a valuable tool in the work-up of all breast abnormalities, both palpable and nonpalpable. The main goal of breast FNA is to give an unequivocal preoperative diagnosis of malignancy in order to allow appropriate patient counseling and definitive clinical management. Equivocal cytological diagnoses should lead to a diagnostic biopsy. The cytological findings should always be evaluated in conjunction with the clinical and radiological findings (triple assessment). Discordant FNA and radiological results usually warrant a diagnostic biopsy.

Rhabdomyosarcoma in the breast has been shown to have variable imaging characteristics, including oval or nodular masses on mammography. Sonographic examination has been shown to demonstrate an inhomogeneous, hypoechoic mass with defined margins and an oval shape, characteristics that are typically considered probably benign. RMS has also been shown to have posterior acoustic enhancement, a finding that has also been described with fibroadenomas [8].

The cytological appearance of the various subtypes of RMS has been addressed in several reports [21–23]. The alveolar RMS shows a predominant small-cell pattern. Dispersed cells are more common than cell clusters or fragments and the cytoplasm is fragile. Stripped nuclei in a blue-gray background of smeared cytoplasm are not uncommon, often demonstrating a “tigroid” appearance. The typical cells resemble small rounded primitive myoblasts with eosinophilic cytoplasm on H&E or grayish blue with MGG staining, with rounded hyperchromatic nuclei and prominent nucleoli. Multinucleated tumor cells are also found [24]. The experience of many investigators has shown that definitive typing of alveolar and embryonal RMS is possible in most cases [14]. It is nevertheless known that the small-cell pattern of RMS may mimic a poorly differentiated carcinoma or a lymphoma [25–27]. The most important diagnostic difficulties lie in distinguishing between the different histological types of small round cell malignant tumors, which include rhabdomyosarcoma, neuroblastoma, the Ewing family of tumors, desmoplastic small round cell sarcoma, and precursor lymphoma/leukemia. The immunocytochemical hallmarks of the various subtypes of RMS are the positive staining with muscle specific actin, desmin, and the specific markers for striated muscle, myogenin, and myoD1. Myoglobin is occasionally present in more differentiated myoblasts. Almost all cases of alveolar RMS present with the chromosomal aberration t (2;13) (q35;q14), resulting in a fusion transcript between the PAX3 and FKHR genes. Cytogenetic investigation of FNA samples to diagnose the typical aberration has been published [28].

The role of FNAC is useful in the clinical setting because it excludes classical carcinoma and identifies unusual lesions that require further investigation, including imaging modalities (MRI/CT). It should, however, be stressed that definitive diagnosis usually relies on core needle biopsy or on surgical tumor excision.

Because fibroadenoma is the most common breast tumor in adolescence, distinguishing early stage RMS from a fibroadenoma is of crucial importance. The knowledge that RMS can very rarely occur in the breast in adults, a high index of suspicion and the identification of the cytological features of small round blue cell tumors aided by immunocytochemical staining can lead to the recognition of this very rare entity and therefore to the correct diagnosis, allowing further imaging to exclude a primary site elsewhere, prior to surgery.

Primary RMS of the breast is very rare, which often leads to delayed histologic confirmation. In our case, as in others in the literature [14, 16, 29, 30], the lesion was preoperatively misdiagnosed as a “carcinoma” of the breast. A core biopsy might well have provided the correct diagnosis preoperatively.

RMS of the breast is an aggressive malignancy. Though the survival in most of the cases is not known, Hays et al.[4] reported it to be between 3 months and 7 years after diagnosis and the 5-year survival rate is 43%.

Our case is unusual, as to the age of presentation in the 7th decade, which is extremely rare, early stage at presentation and adds to the knowledge of this rare tumor entity.

## 4. Conclusion

RMS of the breast is an aggressive malignancy. Although very rare, it has to be thought of, as one of differential diagnoses, particularly in adolescent females. Small round cell malignancy in the breasts of young females should be suspected for the possibility of primary or secondary RMS. Although cytology is efficient in diagnosing small round cell tumors, a high index of suspicion, good knowledge of the cytological criteria in conjunction with clinical and radiological findings and immunochemical stains are prerequisites for the correct diagnosis. Histopathology remains the means for the definitive diagnosis and formulation of treatment plan.

## Figures and Tables

**Table 1:** Cases of primary RMS of the breast in adults: a summary of clinical data.

Reference	Age	Site	Subtype	Size (cm)	LN	Surgical procedure	Other treatment	Prognosis
Italiano, Largillier et al. 2005 [1]	46	Unknown	Embryonal	3.5	NI	Quadrantectomy	CTx, RTx	NED 18 mo

Evans, 1953 [3]	41	Left	Pleomorphic	12	NI	MRM	NI	48 mo

Sailer, 1937 [9]	38	Unknown	Unknown	Unknown	Unknown	“Local removal of the tumor”	Unknown	“shortly afterwards”

Attili, Dadhich et al. 2007 [2]	40	Right	Embryonal	4	+	MRM	CTx	NED 12 mo

Rasinariu, Andreiuolo et al. 2011 [12]	58	Left	Spindle cell	11	NI	Mastectomy	NI	NI

Li, Zhou et al. 2012 [13]	30	Right, then left	Alveolar	2.5	+	MRM	Neo CTx	DOD 29 mo

Bhosale, Kshirsagar et al. 2013 [14]	60	Left	NI	8	+	MRM	CTx	NED 6 mo

Mondal, Mandal et al. 2014 [15]	49	Right	Pleomorphic	7	-	MRM	None	NED 12 mo

Yuan, Hou et al. 2017 [16]	34	Left	NI	3.5	NI	Mastectomy	CTx	NED 23 mo

Trihia et al. 2019 (current)	60	Left	Alveolar	4.8	SLN-	Mastectomy	CTx	NED 24 mo

DOD: died of disease; NED: no evidence of disease; mo: months; NI: not indicated; CTx: chemotherapy; RTx: radiotherapy; MRM: modified radical mastectomy; LN: lymph node status; SLN: sentinel lymph node.
